# Addiction as an Attachment Disorder: White Matter Impairment Is Linked to Increased Negative Affective States in Poly-Drug Use

**DOI:** 10.3389/fnhum.2017.00208

**Published:** 2017-04-28

**Authors:** Human-Friedrich Unterrainer, Michaela Hiebler-Ragger, Karl Koschutnig, Jürgen Fuchshuber, Sebastian Tscheschner, Maria Url, Jolana Wagner-Skacel, Eva Z. Reininghaus, Ilona Papousek, Elisabeth M. Weiss, Andreas Fink

**Affiliations:** ^1^Institute of Psychology, University of GrazGraz, Austria; ^2^Center for Integrative Addiction Research, Grüner Kreis SocietyVienna, Austria; ^3^Department of Psychiatry and Psychotherapeutic Medicine, Medical University GrazGraz, Austria; ^4^Department of Medical Psychology and Psychotherapy, Medical University GrazGraz, Austria

**Keywords:** affectivity, attachment, DTI, spirituality, substance misuse, white matter integrity

## Abstract

Substance use disorders (SUD) have been shown to be linked to various neuronal and behavioral impairments. In this study, we investigate whether there is a connection between the integrity of white matter (WM) and attachment styles as well as different affective states including spirituality in a group of patients diagnosed for poly-drug use disorder (PUD) in comparison to non-clinical controls. A total sample of 59 right-handed men, comprising the groups of patients with PUD (*n* = 19), recreational drug-using individuals (RUC; *n* = 20) as well as non-drug using controls were recruited (NUC; *n* = 20). For the behavioral assessment, we applied the Adult Attachment-Scale, the Affective Neuroscience Personality-Scale (short version) and the Multidimensional Inventory for Religious/Spiritual Well-Being. Diffusion Tensor Imaging was used to investigate differences in WM neural connectivity. Analyses revealed decreased Fractional Anisotropy and decreased Mean Diffusivity in PUD patients as compared to RUC and NUC. No differences were found between RUC and NUC. Additional ROI analyses suggested that WM impairment in the superior longitudinal fasciculus (SLF) and the superior corona radiata (SCR) was linked to more insecure attachment as well as to more negative affectivity. No substantial correlation was observed with spirituality. These findings are mainly limited by the cross-sectional design of the study. However, our preliminary results support the idea of addiction as an attachment disorder, both at neuronal and behavioral levels. Further research might be focused on the changes of insecure attachment patterns in SUD treatment and their correlation with changes in the brain.

## Introduction

Substance use disorders (SUD) have been most prominently described as a chronic, relapsing brain disorder characterized by compulsive drug use (Leshner, 1997; Ersche et al., 2012). In relation to this characterization, numerous studies have linked addictive behaviors to disrupted white matter (WM) and gray matter in the brain (O'Neill et al., 2001; James et al., 2011; Simmons et al., 2012; Batalla et al., 2013). In this study, we will focus on WM (for a general introduction see Bennett and Madden, 2014), since previous research observed poly-drug use disorder (PUD) as being especially harmful for WM (e.g., superior longitudinal fasciculus (SLF) and superior corona radiata (SCR); Unterrainer et al., 2016). Furthermore, there is substantial evidence that heavy substance abuse might be particularly detrimental to the development of WM during adolescence (Lubman et al., 2007; Clark et al., 2012; Baker et al., 2013; Bava et al., 2013).

Based on the evidence for brain affective systems, six distinct emotional circuits were proposed as influencing human personality structure and attachment organization: SEEKING, SADNESS, FEAR, ANGER, CARE, and PLAY (Panksepp et al., 2002; Davis and Panksepp, 2011; Zellner et al., 2011). In studies of SUD, the focus has mostly been on the SEEKING system in order to best describe the haywire primary affective processes that seem to underlie drug cravings (Wright and Panksepp, 2012). Similarly, addictive diseases have been characterized as symptomatic of a deficiency in personality development (Bernstein et al., 1998; Trull et al., 2000) and as an attachment disorder in their own right (Flores, 2001; Schindler and Bröning, 2014).

Previous work in the nascent field of existential neuroscience focused on the emergence of mortality salience (one's own death) and its neuronal correlates (Quirin et al., 2011; Klackl et al., 2012). This work is mainly fueled by the conceptual framework of Terror Management Theory (TMT) (Greenberg et al., 1986), which proposes a basic psychological conflict resulting from having a desire to live while facing death as inevitable. However, in line with classic literature in this field, for instance Martin Heidegger's “Time and Being” Heidegger (1963) or Viktor Frankl's “Man's Search for Meaning “Frankl (1963), we argue that, besides the fear of death and dying, several more facets, such as hope (or despair), forgiveness (or anger and hatred), sense of meaning and connectedness (or fear, alienation) also count as emotions of existential/spiritual relevance. Correspondingly, a lack of spiritual well-being was found to be linked to feelings of alienation and despair in previous research (McClain et al., 2003; Unterrainer and Lewis, 2014). Within the framework of affective neuroscience, spirituality has been named by Davis and Panksepp (2011) as one of the highest human emotions within the pantheon of basic emotions and, moreover, as an important factor for the treatment of addictive diseases.

In this study, we interpret SUD not only as an attachment to an excessive activity (Orford, 2001) but also as an existentially threatening disease, defined by a reduced sense of meaning and of connectedness to the self, other people, or the environment (Nicholson et al., 1994; Wiklund, 2008). As previous research has already linked impaired attachment and personality development to a decreased WM integrity in SUD patients (Unterrainer et al., 2016), we hypothesize that a low level of spiritual well-being might also manifest itself on both behavioral as well as neuronal levels. We primarily investigate the SLF and the SCR by means of Fractional Anisotropy (FA) and Mean Diffusivity (MD) maps since deficiencies in these tracts have been linked to SUD (Bell et al., 2011; Baker et al., 2013; Unterrainer et al., 2016), to impaired decision-making and higher risk-taking behavior (Bechara, 2005; Jacobus et al., 2013), as well as to insecure attachment and personality dysfunctioning (Unterrainer et al., 2016). Furthermore, we expect an increased amount of existential fear and despair as being paralleled by more insecure attachment, higher personality pathology, and decreased spiritual well-being in SUD patients (Unterrainer et al., 2013). In this study, the approach is taken that the severity of drug abuse might follow a continuum which is also reflected on the level of WM integrity: from no/harmless drug use, through continual recreational drug use, up to fully disordered misuse (e.g., Ersche et al., 2013). Therefore, we differentiate between non-drug using (NUC) controls, recreational drug-using (RUC) controls and poly-drug use disordered individuals (PUD). We hypothesize substantial differences between these three groups in neuronal as well as behavioral parameters. This study does not only aim to replicate our prior findings of impaired WM integrity in PUD patients and its relationship to attachment and personality impairments (Unterrainer et al., 2016), but also to follow on those insights by including various affective states as strongly theoretically related with attachment and personality development.

## Materials and methods

### Participants

A total sample of 59 right-handed men between 18 and 35 years of age, composed of one clinical and two non-clinical groups, was investigated. The clinical group (PUD; *n* = 20) was diagnosed for PUD (F19.2) by a licensed psychiatrist according to the International Classification of Diseases version 10 (ICD 10) (Dilling et al., 1991). The first non-clinical group was comprised of nicotine smoking students (RUC; *n* = 20), who reported using illegal substances primarily for recreation at least once a week during the last month. The second non-clinical group was comprised of non-smoking students (NUC; *n* = 20) who reported either no experience with illegal substances or to have tried them just a few times in their life. NUC did not use psychoactive substances in the last 30 days (except for occasional consumption of alcohol). Students were included in the non-clinical groups if they did not report any past or present psychiatric disorder or chronic disease. Psychometric assessment of the clinical subjects took place in two therapeutic facilities of the “Grüner Kreis” society where these participants were undergoing long-term SUD treatment based on the Therapeutic Community concept (De Leon, 2000). The control groups were behaviorally assessed at the University of Graz, Austria. About 90% of the patients living in the therapeutic community are males. All behavioral assessment was conducted via group testing. Written informed consent was acquired from each participant. The study was approved by the ethics committee of the University of Graz, Austria.

### MRI acquisition

Imaging data were attained from a 3T Siemens Skyra (Siemens Medical Systems, Erlangen, Germany) with a 32-channel head coil. The diffusion-weighted structural images were acquired parallel to the anterior-posterior commissure plane using the multi-band sequences (version R012b for Syngo VD13A) provided by the University of Minnesota's Center for Magnetic Resonance Research (https://www.cmrr.umn.edu/multiband/). The parameters were: TR/TE/flip angle = 3036 ms/104 ms/86 deg., matrix size = 96 x 96, FoV = 240 mm, 66 transverse slices of 2.5 mm thickness were measured with no slice gap, multi-band acceleration factor = 3.64 diffusion sensitizing gradient directions were applied (*b* = 2000 s/mm^2^) and one non-diffusion weighted image (*b* = 0 s/mm^2^). Voxel size = 2.5 × 2.5 × 2.5 mm^3^. Two sets of these images were collected for each participant with opposite phase encoding directions [anterior > posterior (A > P) and posterior > anterior (P > A)] so that susceptibility-induced geometric distortions and eddy currents could be corrected using the FSL v5.0 tools TOPUP (Andersson et al., 2003) and eddy (Andersson and Sotiropoulos, 2016). The total acquisition time for these two scans was 7 min.

### Behavioral measures

#### Attachment

Attachment styles were measured by means of the Adult Attachment Scale (AAS) (Collins and Read, 1990). The AAS is based on the assumption that early attachment experiences form a relatively stable inner attachment model that influences individual needs and behavior in later relationships (Bowlby, 1988). It consists of three subscales: Anxiety about being rejected or unloved (Anxiety), comfort with closeness (Closeness) and intimacy and comfort depending on others (Dependence). The German version of the AAS (Schmidt et al., 2004) is comprised of 15 items (5 items per sub-scale). Each item is rated on a 5-point Likert scale ranging from 1 (strongly disagree) to 5 (strongly agree). Cronbach's α were 0.79 for Closeness, 0.72 for Dependence and, 0.78 for Anxiety (Schmidt et al., 2004).

#### Primary emotions

The Brief Affective Neuroscience Personality Scale (BANPS) by Barrett et al. (2013) is the short version of the Affective Neuroscience Personality Scale (ANPS) (Davis et al., 2003). The BANPS measures behavioral traits which are related to Panksepp's concept of basic emotional circuits SEEKING, SADNESS, FEAR, ANGER, CARE, and PLAY (Panksepp, 1998). The questionnaire consists of 33 items rated on a 5-point scale ranging from 1 (strongly disagree) to 5 (strongly agree). In previous research each subscale of the BANPS showed at least a Cronbach's α of 0.70. Additionally, the sub-dimension Spirituality of the long version of the instrument was added for this study, which includes 12 items rated on a 4-point Likert scale (1–“strongly disagree” to 4–“strongly agree”).

#### Religious/spiritual well-being

Religious and spiritual well-being were assessed by means of the Multidimensional Inventory for Religious/Spiritual Well-Being (MI-RSWB) (Unterrainer et al., 2010). The total amount of RSWB refers to “the ability to experience and integrate meaning and purpose in existence through a connectedness with self, others or a power greater than oneself” (Unterrainer et al., 2011) (p. 117). We included the MI-RSWB in order to look separately at the existential as well as on the religious aspects of well-being. The MI-RSWB consists of 48 items that form 6 subscales (8 items per scale; rated on a 6-point Likert scale). These subscales are: Hope Immanent, Forgiveness and Experience of Sense and Meaning as components of an Existential Well-Being (EWB), as well as Hope Transcendent, General Religiosity and Connectedness, as components of a Religious Well-Being (RWB). Each item is rated on a 6-point Likert scale ranging from 1 (“strongly disagree”) to 6 (“strongly agree”). By summing up all six sub-scales a total amount of religious/spiritual well-being (RSBW) can be calculated. In previous studies Cronbach's α was observed to be at least 0.89 for the total score and at least 0.68 for the sub-scales (Unterrainer et al., 2010). In this study, we report the results for EWB, RWB and the total RSWB score.

#### Cognitive ability

Participants also completed the Wonderlic Personnel Test (WPT), a rough screening instrument for the assessment of intelligence (Wonderlic, 1999). This test requires the processing of disordered sentences, analogies, number series, word and sentence comparisons, and geometrical figures within a given time period of 12 min. The WPT contains 50 items with increasing difficulty. The total score is generated from the number of correct responses.

### Statistical analysis

#### MRI data preprocessing

DTI images were processed with FSL 5.0.1 (FMRIB, University of Oxford). Estimation and correction of geometric distortion was carried out with FSL's “top up” and “eddy” using the non-diffusion-weighted images (*b* = 0) collected with each phase encoding direction. After removal of the skull and non-brain tissue using the Brain Extraction Tool (Smith, 2002), the distortion corrected diffusion-weighted images were then used to calculate the diffusion tensors. Using the diffusion tensor information, Fractional Anisotropy (FA) and Mean Diffusivity (MD) maps were computed for each participant using the DTI fit within the Diffusion Toolbox of FSL (FDT). The FA and MD volumes of each participant were brought into a 1 × 1 × 1 mm^3^ common space (Montreal Neurological Institute space; MN1152) via the FMRIB58_FA template using FMRIB's nonlinear registration tool (FNIRT). Then, a mean FA skeleton was created representing the centers of all tracts common to all groups. Individual FA and MD maps were then projected onto this skeleton and finally fed into voxel-wise cross-subject statistics. We used a voxel wise permutation-based (10,000 permutations) statistical approach as implemented in TBSS (Smith et al., 2006). Differences in group contrasts were controlled for mean FA and IQ. Results were corrected for multiple comparisons at *p* < 0.01 using family-wise error (FWE) correction and the threshold-free cluster enhancement (TFCE). The localization of all anatomical information is based on the “JHU ICBM-DTI-81 White-Matter Labels.”

Based on the results of previous research regarding SUD (Bell et al., 2011; Baker et al., 2013; Unterrainer et al., 2016), we further investigated the SLF and the SCR bilaterally. These Regions-of-Interest (ROI) are based on the JHU-ICBM atlas as implemented in FSL 5. We calculated the average FA within each ROI for each subject. This approach avoids the potential disadvantages of the voxel-based methodologies, such as artifacts caused by smoothing techniques or spatial normalization and the need for larger sample sizes (Jones et al., 2005; Niogi and McCandliss, 2006), since these generally make it impossible to distinguish pathological differences in microstructure from those attributable to variability in gross anatomical shape and size.

#### Behavioral data analysis

For group comparisons, one-way analyses of variance were conducted. In addition, Pearson‘s correlation statistics were applied to investigate the relationships between neuronal and behavioral parameters. *Post-hoc* comparisons were accomplished by the Tukey Honest Significant Difference (HSD) test. For comparisons with normative data we performed one-sample *t*-tests. In consideration of the limited sample size and the highly exploratory nature of the study alpha was set to *p* < 0.05 in the behavioral data analysis. To allow a better evaluation of the results we furthermore include a description and interpretation of the effect sizes.

## Results

### Demographics and clinical characteristics

Socio-demographic variables and cognitive ability scores for all three groups are presented in Table 1. In addition, RUC reported a history of nicotine consumption (60% smoked more than 15 cigarettes in the last month) as well as cannabis consumption (70% consumed cannabis at least once in the last month) and occasional use of other psychoactive substances (30%). PUD patients had been in inpatient treatment for a mean time of 23 weeks (*SD* = 11.83) before taking part in the study. They had consumed drugs for about 10.63 years (*SD* = 5.90). Nine PUD patients were in maintenance therapy at the time of the study, while ten were completely drug-free. PUD patients in maintenance therapy received Levomethadone (L-Polamidon) as a substitution agent, with daily doses ranging from 5 to 30 mg. Three PUD patients received psychopharmacological medication (anxiolytic: *n* = 2; hypnotic: *n* = 1; antipsychotic: *n* = 2; antidepressant: *n* = 1). We did not further control for maintenance therapy since previous research revealed no differences in neuronal and behavioral parameters between PUD patients in maintenance therapy and those following self-restraint (Unterrainer et al., 2016).

**Table 1:** **Group differences (ANOVA) in demographic data and cognitive ability**.

**Measures**	**NUC (*n* = 20)**		**RUC (*n* = 20)**		**PUD (*n* = 19)**		***F*_(2, 56)_**	***eta*^2^**	***Post-hoc***
	***M***	***SD***	***M***	***SD***	***M***	***SD***			
Age	23.95	1.91	26.00	2.85	25.84	4.03	2.80	0.09	–
Education (years)	13.50	2.67	14.10	2.94	12.47	2.70	1.71	0.06	–
Education (status)^a^	1.00	0.07	1.00	0.07	0.65	0.07	15.82^**^	0.40	NUC, RUC > PUD
Nicotine consumption^b^	0.00	0.00	3.00	1.49	3.42	1.31	53.02^**^	0.65	NUC < RUC, PUD
WPT	28.00	6.84	29.75	6.44	18.89	7.42	13.79^**^	0.33	NUC, RUC > PUD

** p < 0.01, NUC, Non-using controls, RUC, Recreational using controls, PUD, Poly-drug use disordered patients, WPT, Wonderlic Personnel Test.

a Education status was dummy coded: 0, compulsory education and apprenticeship; 1, qualification for university entrance and university degree.

b *in the last month*.

### Group differences in attachment and personality characteristics

As shown in Table 2, we found several differences with generally large (*eta*^2^ > 0.14) (Cohen, 1990) effect sizes between PUD and the control groups: PUD patients exhibited a higher amount of separation anxiety in relationships (Anxiety) in comparison to NUC and RUC (*p* < 0.01; *eta*^2^ = 0.21). However, we detected no significant differences in the attachment dimensions Dependence and Closeness to others (*p* > 0.05). Compared to normative data (Schmidt et al., 2004) NUC, RUC, and PUD patients showed more Dependence and Closeness to others (*p* < 0.01). However, only PUD patients exhibited increased Anxiety compared to the general population (*p* < 0.01). Furthermore, PUD patients exhibited higher levels of ANGER (*eta*^2^ = 0.14), FEAR (*eta*^2^ = 0.15), and SADNESS (*eta*^2^ = 0.14) compared to the NUC (all *p* < 0.05), while no differences in SEEKING, CARE, or PLAY were found between the three groups (all *p* > 0.05). Notably, RUC, NUC, and PUD patients showed a higher disposition for SEEKING than the general population (*p* < 0.01; Barrett et al., 2013). NUC showed no difference in ANGER to the general population, while RUC (*p* < *0.0*5) and PUD (*p* < 0.01) exhibited a higher ANGER disposition. Only PUD patients showed a higher disposition to FEAR and SADNESS than the general population (*p* < 0.01). A higher amount of PLAY and CARE was observed in all three groups in comparison to the general population (*p* < 0.01). No group differences were found concerning Spirituality, EWB, RWB, and RSWB (all *p* > 0.05). However, a slight trend toward decreased RSWB was observed in PUD patients (*p* = 0.11).

**Table 2:** **Group differences (ANOVA) in behavioral measures**.

**Measures**	**NUC (*n* = 20)**			**RUC (*n* = 20)**		**PUD (*n* = 19)**		***F*_(2, 56)_**	***eta*^2^**	***Post-hoc***
	**α**	***M***	***SD***	***M***	***SD***	***M***	***SD***			
**AAS**										
l.Dependence	0.55	3.81	0.58	3.61	0.58	3.46	0.63	1.68	0.05	–
2.Closeness	0.84	3.92	0.73	3.58	0.89	3.36	1.19	1.56	0.06	–
3.Anxiety	0.81	1.81	0.59	1.92	0.69	2.76	1.14	7.42^**^	0.21	NUC, RUC < PUD
**BANPS**										
4.SEEKING	0.49	3.79	0.59	3.74	0.58	3.72	0.51	0.03	0.00	–
5.ANGER	0.68	2.13	0.48	2.50	0.88	2.84	0.66	4.66^*^	0.14	NUC < PUD
6.FEAR	0.67	2.78	0.60	2.86	0.80	3.44	0.69	4.98^*^	0.15	NUC < PUD
7.SADNESS	0.82	2.24	0.75	2.56	0.83	2.97	0.75	4.38^*^	0.14	NUC < PUD
8.PLAY	0.65	3.82	0.57	3.93	0.66	4.02	0.52	0.57	0.02	-
9.CARE	0.68	3.34	0.35	3.35	0.59	3.46	0.50	0.25	0.01	-
10.Spirituality	0.86	2.26	0.67	2.49	0.85	2.23	0.42	0.87	0.03	-
**MI-RSWB**										
11.EWB	0.75	103.90	14.66	106.70	14.72	100.32	9.36	1.14	0.04	–
12.RWB	0.87	77.45	25.81	80.35	25.81	67.79	13.51	2.09	0.07	–
13.RSWB	0.88	181.35	26.51	187.05	36.11	168.11	18.58	2.31^+^	0.08	–

+ p < 0.11,

* p < 0.05,

** *p < 0.01, ?, Cronbach Alpha; NUC, Non-using controls; RUC =, Recreational using controls; PUD, Poly-drug use disordered patients; AAS, Adult Attachment Scale; BANPS, Brief form of the Affective Neuroscience Personality Scale; MI-RSWB, Multidimensional Inventory for Religious and Spiritual Well-Being; EWB, Existential Well-Being; RWB, Religious Well-Being; RSWB, Religious/Spiritual Well-Being*.

### Intercorrelations of attachment and personality characteristics

As depicted in Table 3, we found negative correlations of small to medium strength (0.20 < *r* < *0.50*) (Cohen, 1990) between parameters of attachment security and SADNESS as well as FEAR while anxious attachment showed medium (*r* > *0.30*) to strong (*r* > *0.50*) positive correlations with SADNESS, FEAR, and ANGER (all *p* < 0.05). Moreover, we observed a medium negative association between EWB and ANGER (*p* < 0.05). Notably, CARE was the only parameter that showed small to medium positive correlations with all parameters of spirituality (all *p* < 0.05).

**Table 3:** ***Intercorrelations for behavioral measures***.

**Variable**	**1**	**2**	**3**	**4**	**5**	**6**	**7**	**8**	**9**	**10**	**11**	**12**	**13**
**AAS**													
1. Dependence		0.52^**^	0.68^**^	−0.09	−0.23	−0.34^**^	−0.27^*^	0.19	0.29^*^	0.06	0.03	0.03	0.12
2. Closeness			−0.56^**^	−0.01	−0.21	−0.43^**^	−0.33^**^	0.21	0.28	0.17	0.19	0.07	0.14
3. Anxiety				0.18	0.34^**^	0.51^**^	0.39^**^	0.15	−0.12	0.09	−0.09	0.07	0.01
**BANPS**													
4. SEEKING					−0.10	0.06	0.05	0.13	−0.02	−0.06	0.08	0.00	0.04
5. ANGER						0.24	0.28^*^	0.01	0.10	−0.07	−0.31^*^	−0.11	−0.23
6. SADNESS							0.55	0.07	−0.15	0.07	0.07	−0.02	0.04
7. FEAR								−0.04	−0.08	−0.19	−0.14	−0.25	−0.24
8. CARE									0.13	0.34^**^	0.31^*^	0.29^*^	0.34^**^
9. PLAY										−0.09	−0.04	−0.14	−0.11
10. Spirituality											0.47^**^	0.72^**^	0.73^**^
**MI-RSWB**													
11. EWB												0.44^*^	0.77^**^
12. RWB													0.91^**^
13. RSWB													

* p < 0.05,

** *p < 0.01. AAS, Adult Attachment Scale, BANPS, Brief form of the Affective Neuroscience Personality Scale, MI-RSWB, Multidimensional Inventory for Religious and Spiritual Well-Being. EWB, Existential Well-Being, RWB, Religious Well-Being, RSWB, Religious/Spiritual Well-Being*.

### Whole-brain analysis of white matter integrity

**Figure 1:**
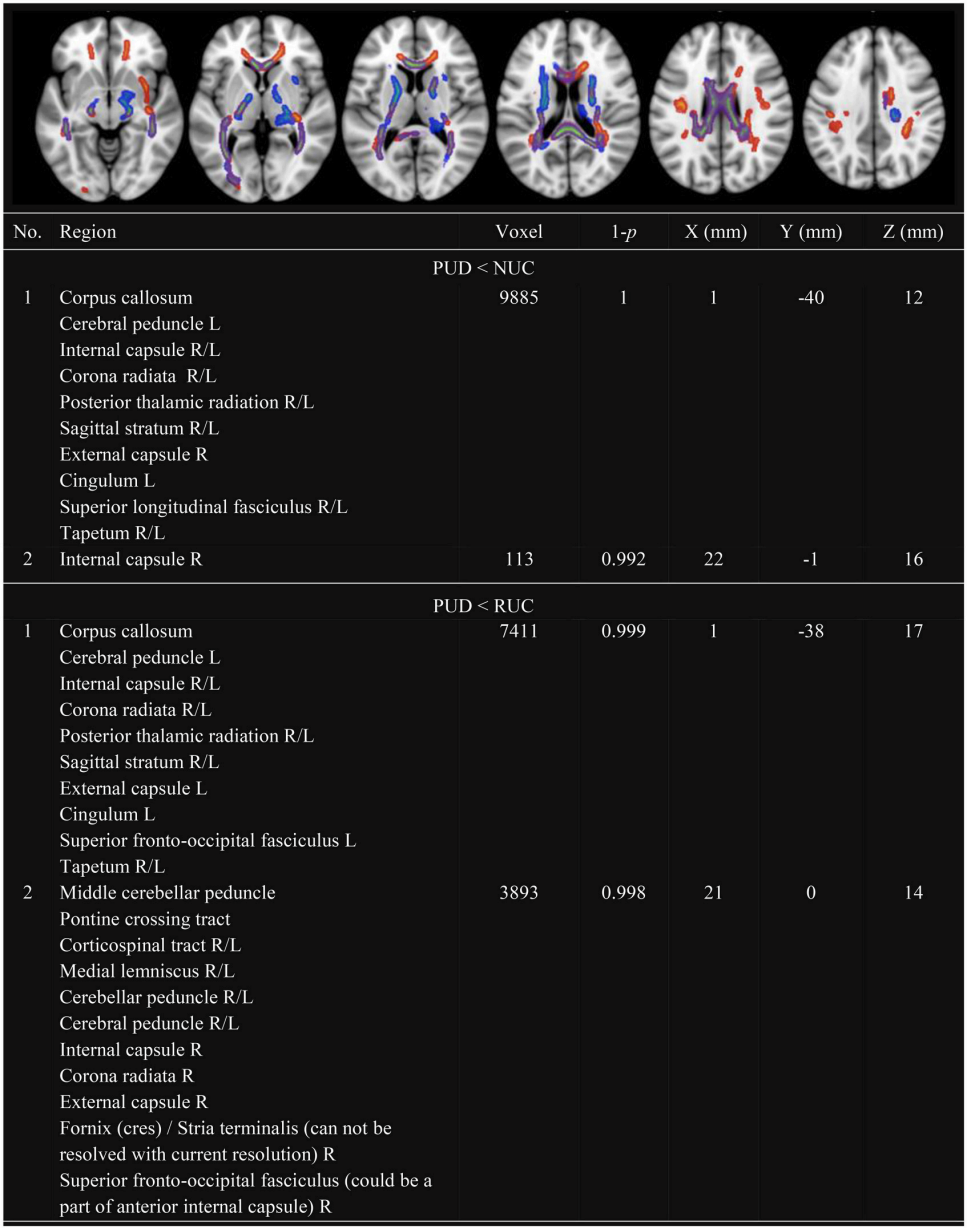
**Clusters with decreased FA in PUD compared to NUC and RUC**. Only Clusters with a size more than 100 voxel are presented. FA, Fractional Anisotropy; NUC, Non-using controls; RUC, Recreational using controls; PUD, Poly-drug use disordered patients; PUD < NUC, Decreased FA in PUD in comparison to NUC (red-yellow); PUD < RUC, Decreased FA in PUD in comparison to RUC (blue-green); No., Number; Region, Included regions according to JHU ICBM-DTI-81 White-Matter Labels; R, Right; L, Left; Voxel; Number of voxel per cluster; 1-*p*, Statistical peak-value for each cluster; X–Y–Z (mm) = Peak-coordinates for each cluster. Significantly different clusters have been thickened with the FMRIB Software Library option “tbss_fill.”

**Figure 2:**
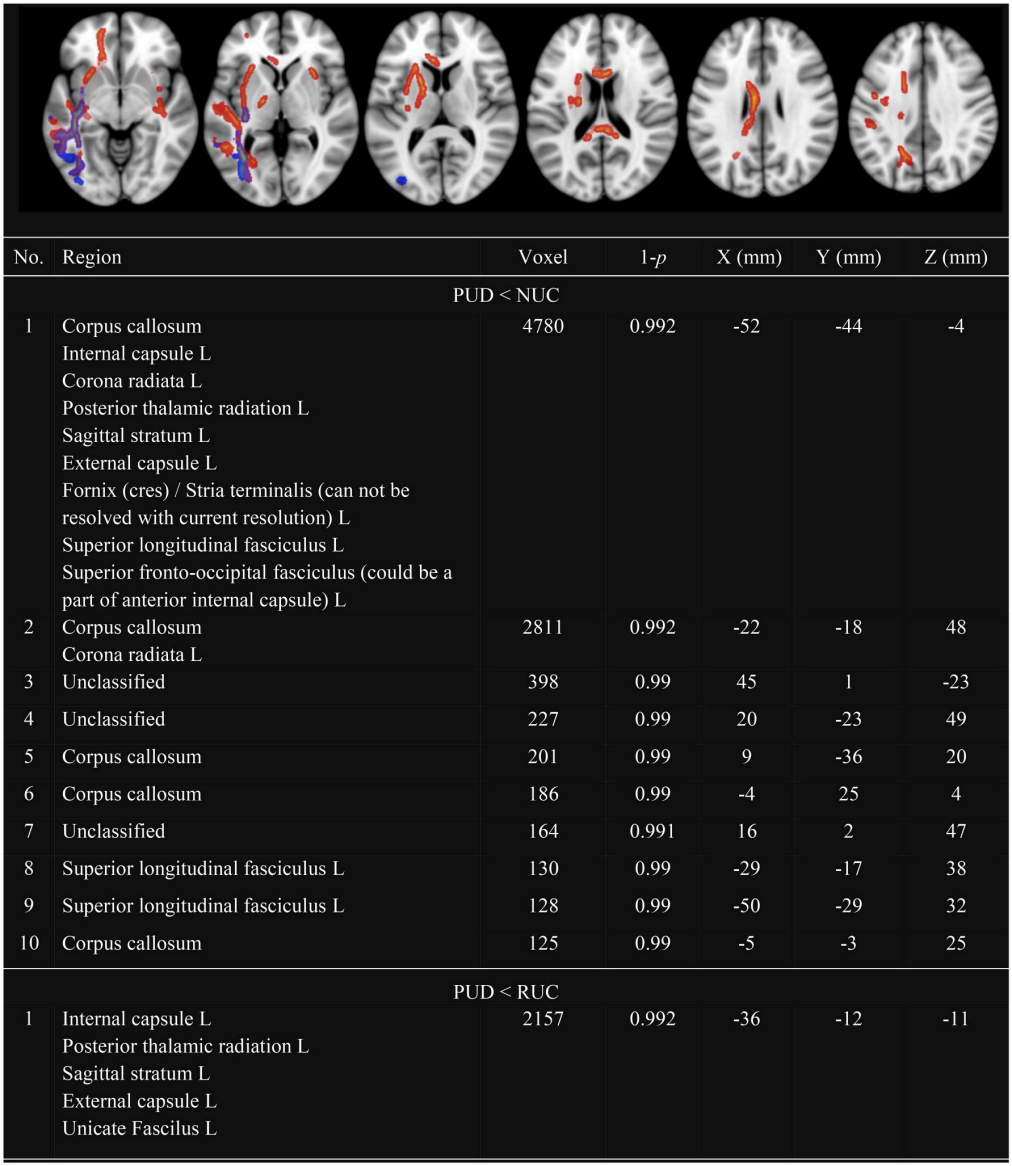
**Clusters with decreased MD in PUD compared to NUC and RUC**. Only Clusters with a size more than 100 voxel are presented. MD, Mean Diffusivity; NUC, Non-using controls; RUC, Recreational using controls; PUD, Poly-drug use disordered patients; PUD < NUC, Decreased MD in PUD in comparison to NUC (red-yellow); PUD < RUC, Decreased MD in PUD in comparison to RUC (blue-green); No., Number; Region, Included regions according to JHU ICBM-DTI-81 White-Matter Labels; R, Right; L, Left; Voxel, Number of voxel per cluster; 1-*p* = Statistical peak-value for each cluster; X –Y– Z (mm) = Peak-coordinates for each cluster. Significantly different clusters have been thickened with the FMRIB Software Library option “tbss_fill.”

In line with previous research, we observed several clusters with decreased FA (see Figure 1) and decreased MD (see Figure 2) in PUD patients compared to NUC and RUC. There were no regions in which PUD patients showed increased FA or increased MD compared to NUC and RUC. Interestingly, no differences in FA or MD were observed between NUC and RUC.

Specifically, as shown in Figure 1, clusters with decreased FA in PUD patients involved the following WM tracts in both hemispheres: corpus callosum, corona radiata, SLF, internal capsule, posterior thalamic radiation, sagittal stratum, and tapetum. Another large cluster, observed in the divergence between PUD patients and RUC, included the corticospinal tract, the medial lemniscus, the cerebellar peduncle and the cerebral peduncle of both hemispheres.

With respect to MD (see Figure 2), differences were observed almost exclusively in the left hemisphere. Clusters with decreased MD in PUD patients involved the corpus callosum as well as: corona radiata, SLF, internal and external capsule, posterior thalamic radiation and sagittal stratum. These differences were more pronounced between NUC and PUD patients than between RUC and PUD patients.

### Regions-of-interest analysis

In order to further explore group differences and possible connections between WM integrity and behavioral measures, we conducted an ROI analysis in the SLF and the SCR. We focused on FA, the most widely used DTI parameter (Smith et al., 2006), to reduce the possibility of an inflated alpha-error due to multiple comparisons.

#### Group comparison

As shown in Table 4, we found several relevant differences in the ROIs between the three groups of NUC, RUC and PUD patients, assessed by univariate General Linear Models (with the factor GROUP). We observed an increased amount of FA in the left (*p* < 0.01; *eta*^2^ = 0.15) and the right (*p* < 0.05; *eta*^2^ = 0.22) SLF of NUC and RUC in comparison to PUD patients. Moreover, NUC showed increased FA in the left (*eta*^2^ = 0.16) and right (*eta*^2^ = 0.14) SCR (*p* < 0.05).

**Table 4:** **Group differences in FA in Regions-of-Interest (ROI)**.

**Measure**	**NUC**		**RUC**		**PUD**		***F*_(2, 59)_**	***eta*^2^**	***Post-ho*c**
	***M***	***SD***	***M***	***SD***	***M***	***SD***			
SLF_R	0.458	0.020	0.456	0.027	0.436	0.024	4.86^*^	0.15	NUC, RUC > PUD
SLF_L	0.448	0.019	0.444	0.021	0.423	0.022	8.12^**^	0.22	NUC, RUC > PUD
SCR_R	0.450	0.025	0.447	0.019	0.429	0.025	4.46^*^	0.14	NUC > PUD
SCR_L	0.455	0.021	0.449	0.023	0.431	0.026	5.27^*^	0.16	NUC > PUD

* p<.05,

** *p<.01, FA, Fractional Anisotropy; NUC, Non-using controls; RUC, Recreational using controls; PUD, Poly drug use disordered patients; FLS, Fasciculus longitudinalis superior; CRS, Corona radiata superior; L, Left; R, Right*.

#### The relationship between FA and behavioral measures in PUD patients

The observed correlations between neuro measures and personality variables were generally medium (*r* > 0.30) or large (*r* > 0.50) (Cohen, 1990). Although severely restrictive testing (i.e., the use of Bonferroni correction) would considerably diminish the number of significant results, the overall pattern of the correlations appears to be quite interesting to note. As shown in Table 5, FEAR showed a medium negative correlation with FA in the right SCR (*r* = −0.46; *p* < 0.05) and a medium negative correlation trend with FA in the right SFL (*r* = −0.44; *p* = *0.0*6). For CARE we observed a large positive correlation with FA in the left SFL (*r* = 0.50; *p* < 0.05) while PLAY showed a medium negative correlation trend with FA in this ROI (*r* = −0.39; *p* = 0.10). For SADNESS there was a medium negative correlation trend with FA in the right SCR (*r* = −0.40; *p* = 0.09). Regarding the attachment dimensions, Dependence showed a strong positive correlation with FA in the right SCR (*r* = 0.58; *p* < 0.01) and a medium positive correlation trend with FA in the left SCR (*r* = 0.41; *p* = 0.09). Anxiety showed a significant negative correlation trend with FA in the right SCR (*p* = 0.11). Finally, there were medium positive correlation trends between EWB and FA in the left and right SFL (both *r* = 0.44; *p* = 0.06). This pattern of correlations seems to be common for PUD patients as we did not find a similar pattern for RUC or NUC. Since the reported correlations are based on a sample of only 19 patients, these analyses are highly exploratory and await replication in future, more powerful studies.

**Table 5:** **Correlations of behavioral measures with FA in the selected ROIs for PUD (*n* = 19)**.

**Measures**	**SLF_R**	**SLF_L**	**SCR_R**	**SCR_L**
**AAS**				
1.Dependence	0.13	0.14	0.58^**^	0.41^+^
2.Closeness	0.07	−0.14	0.22	−0.07
3.Anxiety	−0.02	0.09	−0.39^+^	−0.08
**BANPS**				
4.SEEKING	0.04	0.19	0.13	0.26
5.ANGER	−0.09	−0.20	0.21	0.23
6.SADNESS	−0.18	−0.01	−0.40^+^	−0.22
7.FEAR	−0.44^+^	−0.25	−0.46^*^	−0.38
8.CARE	0.28	0.50^*^	0.30	0.23
9.PLAY	−0.25	−0.39^+^	−0.19	−0.31
10.Spirituality	0.21	−0.05	0.02	−0.04
**MI-RSWB**				
11.EWB	0.44^+^	0.44^+^	0.06	0.16
12.RWB	0.06	0.02	−0.15	−0.06
13.RSWB	0.27	0.24	−0.08	0.04

+ p < 0.11,

* p < 0.05,

** *p < 0.01; FA, Fractional Anisotropy; PUD, Poly-drug use disordered patients; SLF, Superior longitunidal fasciculus; SCR, Superior corona radiata; L, Left; R, Right; AAS, Adult Attachment Scale; BANPS, Brief form of the Neuro-Affective Personality Scale; MI-RSWB, Multidimensional Inventory for Religious and Spiritual Well-Being; EWB, Existential Well-Being; RWB, Religious Well-Being; RSWB, Religious/Spiritual Well-Being*.

## Discussion

In this study, we further investigated the relationship between WM integrity and behavioral parameters of attachment and personality in PUD patients compared to two different (recreational drug-use [RUC] vs. non-drug using [NUC]) control groups. Our results confirm previous findings of increased attachment pathology in PUD patients (Unterrainer et al., 2016; Hiebler-Ragger et al., 2016b) as being related to diminished WM integrity in distinct areas (Unterrainer et al., 2016). By enhancing previous research we observed affective states, such as SADNESS or FEAR tend to related to impaired WM. This finding could be further investigated as negative emotional states, such as anger, anxiety, depression, frustration, and boredom, were observed to be associated with the highest rate of relapse (Larimer and Palmer, 1999). In line with relevant literature (Moeller et al., 2005; Schindler and Bröning, 2014), PUD patients exhibited a higher amount of anxious attachment as well as more ANGER, FEAR, and SADNESS than NUC. Notably, we did not find any differences in SEEKING, which has been discussed in previous work as being pathologically abridged in SUD (Alcaro and Panksepp, 2011; Wright and Panksepp, 2012). A possible explanation for this finding is provided by the fact that the PUD patients lived in the environment of a therapeutic community (De Leon, 2000; Chiesa and Fonagy, 2010) when participating in this study. This environment might act as a substitution agent for the PUD patients thereby blocking the abridged SEEKING dimension. Otherwise, abridged SEEKING might increase drug craving and the possibility of relapse (Drummond, 2001; Alcaro and Panksepp, 2011). Since SUD was also discussed as being almost the opposite to depression in psychiatric disorders (Zellner et al., 2011), the increased amount of SADNESS in PUD patients might also be linked to a potentially blocked seeking system.

Additionally, in this study, we sought to put our findings in a broader, clinical context by relating neuroscientific findings to affective states, such as existential and spiritual well-being (Inzlicht et al., 2011). In contrast to our assumption (Unterrainer et al., 2013), we did not observe a decreased EWB in the PUD patients. Furthermore, we observed no differences in parameters of spirituality between PUD patients and both RUC and NUC. However, it should be noted that there is at least a tendency for decreased Religious/Spiritual Well-Being in PUD patients (*p* = 0.11), which is in line with previous work where substantial differences between SUD patients and healthy controls are documented (Kendler et al., 2003; Unterrainer et al., 2013).

Recent research also indicates an increased amount of insecure attachment as a significant predictor of mood pathology. However, this relationship seems to be moderated by EWB (Hiebler-Ragger et al., 2016a). Therefore, in this study, the tentative links between attachment, affective states (including RSWB) and WM integrity in PUD patients might point to a close common neuronal ground. From a clinical perspective, these findings support the idea of considering religious/spiritual aspects in addiction treatment in an effort to facilitate more secure attachment experiences and thereby promote the ability for a better regulation of affective states (Unterrainer et al., 2013). Especially the setting of a therapeutic community (De Leon, 2000), a long-term, caregiving and drug-free inpatient treatment, aims to facilitate corrective emotional experiences, which might act as a fertile soil for the post-maturation of initially insecure attachment patterns (Flores, 2001). In addition, we observed a positive relationship between all parameters of spirituality and CARE, which indicates stronger pro-social values (Piedmont, 2004). This finding might be further explored in the future as there is some evidence that religion and spirituality, as a method of finding meaning, are linked to certain areas in the brain (Inzlicht et al., 2011).

## Limitations and future perspectives

In this study, we sought to pinpoint neuronal correlates of attachment and personality pathology in PUD in order to enhance our understanding of SUD. Further research might also benefit from focusing on neuroplasticity in the course of SUD treatment. Furthermore, in this study, we assumed the hypothesis of a continuum in SUD, with addiction at its high end. This hypothesis, however, was only partly confirmed by our findings, as no imaging differences were found between the RUC and the NUC groups. Especially the non-clinical subjects could be assessed more precisely by including validated measures of life time substance use (Czermak et al., 2005). Moreover, due to the small sample size we could not control for several socio-anamnestic variables as well as other potentially confounding factors, such as for instance total brain volume or data processing methods. Furthermore, we underline that our findings still have to be taken as highly explorative and largely need to be confirmed in future research.

Finally, we confer with Sullivan and Hagen (2002) in saying that humans have always shared a co-evolutionary relationship with psychotropic substances. However, at a certain point the use of substances gets out of control and leads to severe existentially threatening psychiatric and neurological disorders. Either way, based on these preliminary findings we support the notion (Chung et al., 2013) that a neuro-scientifically informed approach that considers the role of attachment and affective states (including spirituality) enriches the ways of relating to patients in SUD-treatment.

## Ethics statement

This study was carried out in accordance with the recommendations of the ethics committee of the University of Graz, Austria with written informed consent from all subjects. All subjects gave written informed consent in accordance with the Declaration of Helsinki. The protocol was approved by the ethics committee of the University of Graz, Austria.

## Author contributions

HU and AF conceptualized the study. MH, KK, JF, ST, MU, and JW collected, analyzed and interpreted the data. HU and MH drafted the manuscript. ER, IP, EW, and AF critically reviewed the manuscript. All authors gave their final approval of the manuscript.

### Conflict of interest statement

The authors declare that the research was conducted in the absence of any commercial or financial relationships that could be construed as a potential conflict of interest.
